# Bcl-2 and FAS as Apoptosis-Related Markers in Patients with Convulsive Status Epilepticus

**DOI:** 10.3390/jcm14196734

**Published:** 2025-09-24

**Authors:** Lejla Ćorić, Slavica Sović, Brankica Šimac, Iva Mihaljević, Ines Vukasović, Zrinka Čolak Romić, Ivana Šušak Sporiš, Željka Petelin Gadže

**Affiliations:** 1 Department of Neurology, Clinical Hospital Dubrava, Referral Centre of the Ministry of Health of the Republic of Croatia for Preoperative Treatment of Patients with Pharmacoresistant Epilepsy, Avenija Gojka Šuška 6, 10000 Zagreb, Croatia; iva_mihaljevic@hotmail.com (I.M.); zrc0410@gmail.com (Z.Č.R.); isusak1406@gmail.com (I.Š.S.); 2 Medical Faculty, University of Zagreb, Šalata 3b, 10000 Zagreb, Croatia; slavica.sovic@gmail.com (S.S.); zeljka.petelin@mef.hr (Ž.P.G.); 3 Department of Clinical Chemistry, Clinical Hospital Dubrava, Avenija Gojka Šuška 6, 10000 Zagreb, Croatia; brankica.simac@gmail.com; 4 Department of Clinical Chemistry, University Hospital Centre Sestre Milosrdnice, Vinogradska Cesta 26, 10000 Zagreb, Croatia; ines.vukasovic@gmail.com; 5 Department of Neurology, University Hospital Centre Zagreb, Referral Centre of the Ministry of Health of the Republic of Croatia for Epilepsy, Affiliated Member of the ERN EpiCARE, Kišpatićeva 12, 10000 Zagreb, Croatia

**Keywords:** status epilepticus, apoptosis, Bcl-2, FAS, programmed cell death

## Abstract

**Background**: Status epilepticus (SE) is a neurological emergency associated with neuronal injury and activation of apoptotic pathways. While these mechanisms are well described in experimental models, evidence in humans is limited. This study evaluated Bcl-2 and FAS—key apoptosis-related proteins—in the serum and cerebrospinal fluid (CSF) of patients with convulsive SE. **Methods**: Between February 2024 and January 2025, CSF and serum samples were collected from 18 adults with convulsive SE within 48 h of onset, and from 15 control subjects. Patients with acute brain injury, stroke, tumors, or central nervous system infections were excluded. Bcl-2 and FAS concentrations were quantified using ELISA. Serum samples were obtained at diagnosis (S1), 24 h (S2), and 7 days (S3). **Results**: CSF Bcl-2 levels were significantly higher in SE patients compared with controls (z = 4.1, *p* < 0.001). CSF FAS levels did not differ significantly (z = 0.07, *p* = 0.94). No differences in serum Bcl-2 were observed. In contrast, serum FAS concentrations were significantly elevated at all three time points in SE patients compared with controls (S1–S3; all *p* < 0.001). **Conclusions**: Convulsive SE is associated with distinct apoptotic responses in the central nervous system and periphery. Elevated CSF Bcl-2 may reflect acute neuroprotective or stress-related responses, whereas persistently increased serum FAS suggests systemic apoptotic activation. These findings highlight the potential prognostic and therapeutic relevance of apoptosis-related biomarkers in SE.

## 1. Introduction

Status epilepticus (SE) is the most severe form of seizures and is linked to high rates of morbidity and mortality [1]. With better access to electroencephalography (EEG), the implementation of new diagnostic criteria, and an aging population, the incidence of this condition is increasing [2,3]. Despite advancements in treatment, the mortality rate has remained unchanged over the past 30 years [4,5]. Unfortunately, patients who survive often develop epilepsy, encephalopathy, and functional disability [6].

In 2015, the operational definition provided by the International League Against Epilepsy (ILAE) described SE as a condition resulting from the failure of mechanisms responsible for seizure termination or the activation of mechanisms that lead to prolonged seizure activity, which may ultimately cause neuronal injury and death [7]. Although some acute changes in brain tissue are reversible, some patients with SE develop diffuse and/or focal brain atrophy. However, it remains unclear whether this atrophy is solely a result of SE itself or related to prolonged treatment and/or complications [8,9].

Most information about the pathophysiological mechanisms of neuronal injury during SE has been obtained from decades of research using animal models [10]. Current findings suggest that apoptosis, necroptosis, pyroptosis, ferroptosis, and autophagy—pathways of regulated cell death—are responsible for neuronal injury [6]. However, studies using SE models have also shown inconsistent results regarding the relationship, prevalence, and dominance of specific biochemical mechanisms, indicating variability in how epileptic activity affects brain cells [6,11].

Some features of apoptosis have been observed in brain cells of SE models and in pathological and histological studies of patients with temporal lobe epilepsy (TLE) [6,12]. Apoptosis can proceed via two main routes: the extrinsic pathway, initiated by death receptors, and the intrinsic pathway, regulated by mitochondrial integrity [13]. The extrinsic pathway is triggered when ligands bind to membrane-bound receptors such as TNFR1, FAS (CD95), or DR4 (TRAIL receptor 1), thus activating caspase-dependent cascades [13,14]. Conversely, the intrinsic pathway is induced by intracellular disturbances like calcium overload, excessive reactive oxygen species (ROS), or dysregulation of Bcl-2 family proteins, ultimately causing mitochondrial dysfunction and cytochrome c release [15,16].

Within these frameworks, FAS and Bcl-2 are prominent and well-studied markers of extrinsic and intrinsic apoptotic regulation, respectively. The death receptor FAS has been implicated as a key mediator of apoptosis in both normal and pathological contexts [13,14]. In experimental epilepsy models, SE has been shown to significantly increase Fas and FasL expression in the hippocampus, accompanied by caspase-3 activation—findings consistent with activation of the extrinsic apoptotic pathway [17]. Similarly, Bcl-2, a prototypical anti-apoptotic protein located in the inner mitochondrial membrane, helps stabilize mitochondrial function and prevents the release of cytochrome c [12,13,18]. Although other Bcl-2 family proteins like Bcl-xL and Mcl-1 are also important regulators, Bcl-2 remains the standard and most extensively characterized member, serving as a reference in mitochondrial apoptosis research [13,19,20].

Overall, evaluating FAS (extrinsic) and Bcl-2 (intrinsic) together provides a mechanistic framework for studying the two primary apoptotic pathways potentially activated during SE. Despite their conceptual importance, the roles of these factors in human SE are still not well understood. Most current studies have been limited to experimental models or other neurological diseases, creating a significant gap in translational knowledge [14,15,16,19,21].

Given their potential as markers of neuronal injury, we aimed to assess the detectability and clinical relevance of FAS and Bcl-2 in the cerebrospinal fluid (CSF) and serum of patients with convulsive status epilepticus (CSE). CSF biomarkers provide clear advantages over serum, as they more directly reflect processes specific to the central nervous system (CNS), whereas serum levels can be influenced by systemic inflammation and peripheral immune responses [17,18,22,23].

We hypothesized that higher CSF Bcl-2 levels would indicate adaptive neuroprotective mechanisms and be associated with better outcomes. By comparing CSF and serum profiles, this study aims to differentiate brain-specific apoptotic activity from systemic processes and to determine whether FAS and Bcl-2 can serve as meaningful clinical biomarkers in SE.

## 2. Materials and Methods

### 2.1. Study Design and Participants

In this prospective study, serum and cerebrospinal fluid (CSF) samples were collected from 18 patients with CSE and 15 control subjects treated at the Department of Neurology, Clinical Hospital Dubrava, Zagreb, Croatia, between February 2024 and January 2025.

The inclusion criteria for the SE group were (1) aged ≥ 18 years and (2) a diagnosis of CSE established through clear clinical history and assessment according to the ILAE definitions [7]. The patients with generalized convulsive (GCSE), focal onset evolving into bilateral CSE, of unknown whether focal or generalized onset SE, were included. Additionally, SE was considered ongoing when seizures were clinically ended, but the patient remained comatose, and an EEG indicated ongoing electrical seizure activity (CSE to nonconvulsive SE).

Exclusion criteria were pregnancy, diagnosis of posthypoxic–myoclonic SE, acute brain injury, acute stroke, central nervous system (CNS) infection, malignancy, hepatic and uremic encephalopathy.

**Figure 1:**
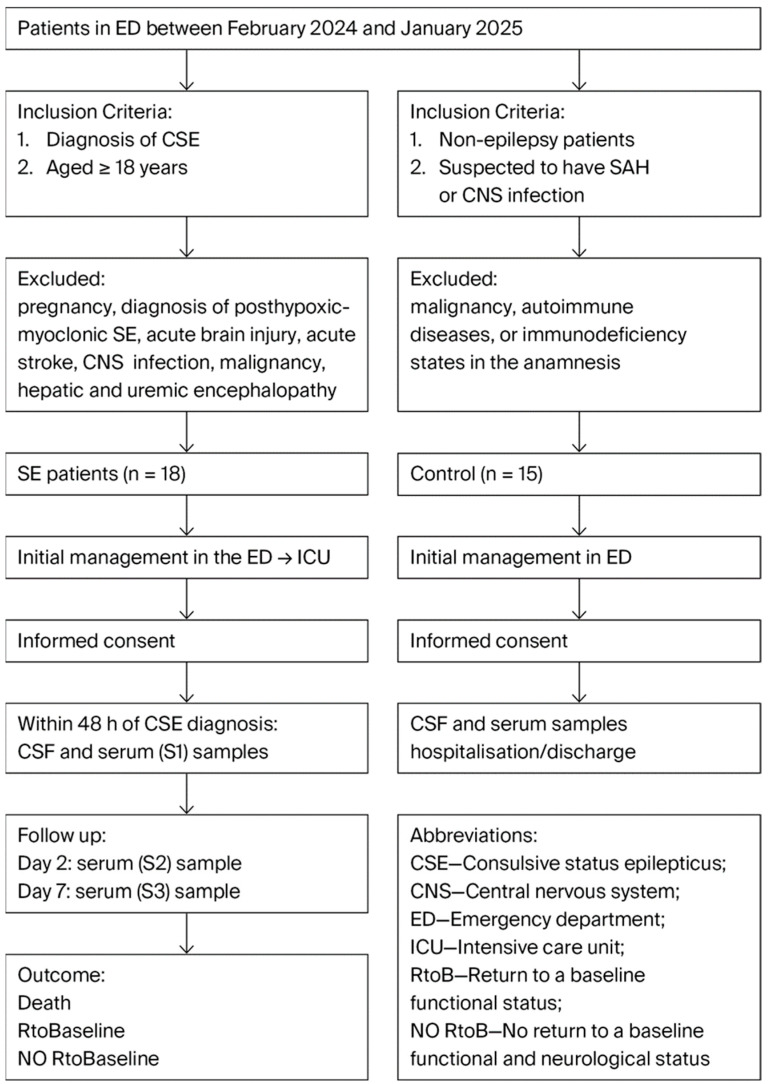
Diagram of patients’ flow and sampling points.

The control group comprised 15 patients who underwent lumbar puncture to exclude CNS infection or subarachnoidal hemorrhage; all of them were without malignancy, autoimmune diseases, or immunodeficiency states in the anamnesis (Figure 1: Diagram of patients’ flow and sampling points).

### 2.2. Procedure

#### 2.2.1. Patients’ Clinical Data

Demographic and clinical data were prospectively collected using a predefined dataset: patients’ age, sex, previous epilepsy diagnoses or seizures, previous history of stroke, alcohol dependence, and previous history of traumatic brain injury (TBI). SE semiology and etiology were classified according to the ILAE classification system [7]. Patients with SE underwent an EEG examination (NihonKohden, 21 channels, International 10–20 System; Polaris ONE V.4.0.3.0). EEG findings were classified according to the current terminology of the American Clinical Neurophysiology Society Consensus and Salzburg’s Consensus EEG Criteria [24,25].

Clinical outcomes were defined as return to a baseline functional status (RtoB), no return to a baseline functional and neurological status (NO RtoB), and death (in-hospital mortality).

The included patients were initially treated in the emergency department and then admitted to the neurological intensive care unit (ICU). Diagnostic procedures and treatment were conducted independently, following current clinical guidelines.

CSF examination and computed tomography/magnetic resonance imaging of the brain were performed to determine the aetiology of SE.

CSF and initial serum samples (S1) were collected no later than 48 h after SE diagnosis. Subsequent serum samples were collected 24 h (S2) and 7 days (S3) after the initial sampling.

The following laboratory data, within the first 48 h, at the earliest feasible time for testing, were also collected from all participants: CSF fluid lactate (mmol/L), CSF glucose level (mmol/L), CSF protein level(g/L), serum values of C-reactive protein (CRP; mg/L), and white blood cell count (WBC).

#### 2.2.2. Serum and CSF Apoptosis Markers Analysis

The sample collection included serum and CSF from each participant. All samples were immediately sent to the laboratory for processing. The blood samples were centrifuged for 10 min at 2000 rpm.

The CSF and serum aliquots were stored at −80 °C until analysis. Levels of Bcl-2 and FAS in serum and CSF were measured using commercially available enzyme-linked immunosorbent assay (ELISA) kits (cat.no. NBP1-91188 for Bcl-2, cat.no. NBP1-91190 for FAS; Novus Biologicals, Centennial, CO, USA) on the BEP 2000 Advance^®^ System (Siemens Healthcare Diagnostics, GmbH, Marburg, Germany). Samples were tested according to the manufacturer’s instructions and the recommended dilutions. The detection ranges were 0.5–32 ng/mL for Bcl-2 and 15.6–1000 ng/mL for FAS.

### 2.3. Standard Protocol Approval

The study protocol was approved by the Institutional Ethics Committee of our Clinical Hospital (ethics committee approval number 2023/1602-01, date: 16 February 2023) and was conducted according to the ethical principles for medical research involving human subjects, according to the Declaration of Helsinki. Patients or their legally authorized representatives or relatives were notified about enrollment in the study as soon as possible, usually while the patient was in the emergency department. Informed consent was obtained from all participants or their relatives.

### 2.4. Statistical Analysis

Statistical package program Statistica StatSoft25.0TIBCO Software Inc. (San Ramon, CA, USA), was used for statistical analysis. Differences in the distributions of numerical variables between SE patients and the control group were assessed using the Mann–Whitney U test. Categorical variables were compared using Pearson’s chi-square (χ^2^) test. To evaluate changes in the distribution of FAS and Bcl-2 marker levels at three different time points, the Friedman test for repeated measures was employed.

Statistical significance was set at *p* < 0.05.

### 2.5. Outcomes

The primary endpoint was to identify apoptosis markers in patients with convulsive SE by analyzing CSF samples during the acute phase of this life-threatening condition, as well as serum samples, which were repeated for each patient at different time points from the onset of the seizures.

Secondary endpoints were to determine whether there is a correlation between the level of apoptosis markers and unfavorable treatment outcomes. (Supplementary Materials Tables S1 and S2)

## 3. Results

### 3.1. Patient Features

Samples of serum and cerebrospinal fluid were obtained from 18 patients (10 male and 8 female) and analyzed. Six patients had a previously established diagnosis of epilepsy, three patients had a previous history of traumatic brain injury, five patients had suffered a stroke, and six patients had a history of alcohol abuse. (Supplementary Materials, Table S1)

All patients were admitted to the ICU, and 6 patients required mechanical ventilation for the administration of third-line therapy. Of the 18 patients, 4 died at the end of treatment, and of the 14 survivors, 6 experienced a decline in functional capacity. Neuroimaging (CT and MRI) revealed peri-ictal changes in three patients (Table 1).

### 3.2. Data Analysis

**Figure 2:**
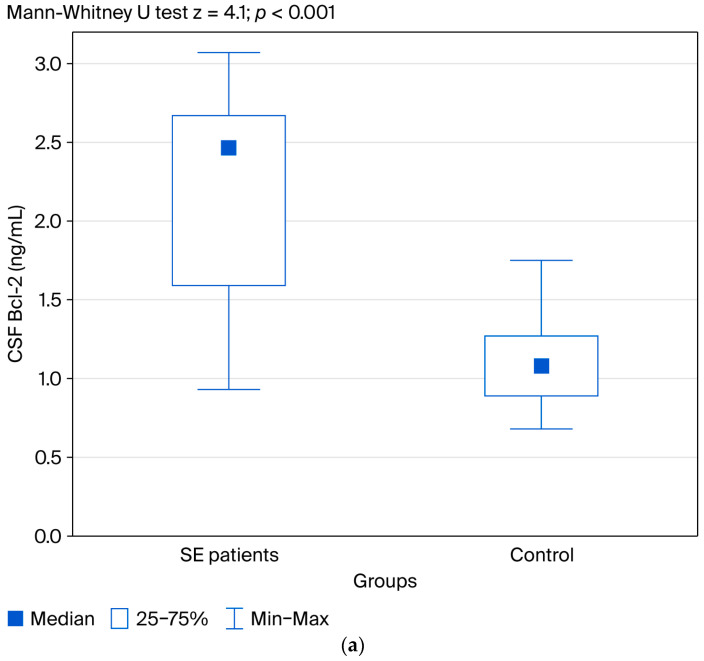

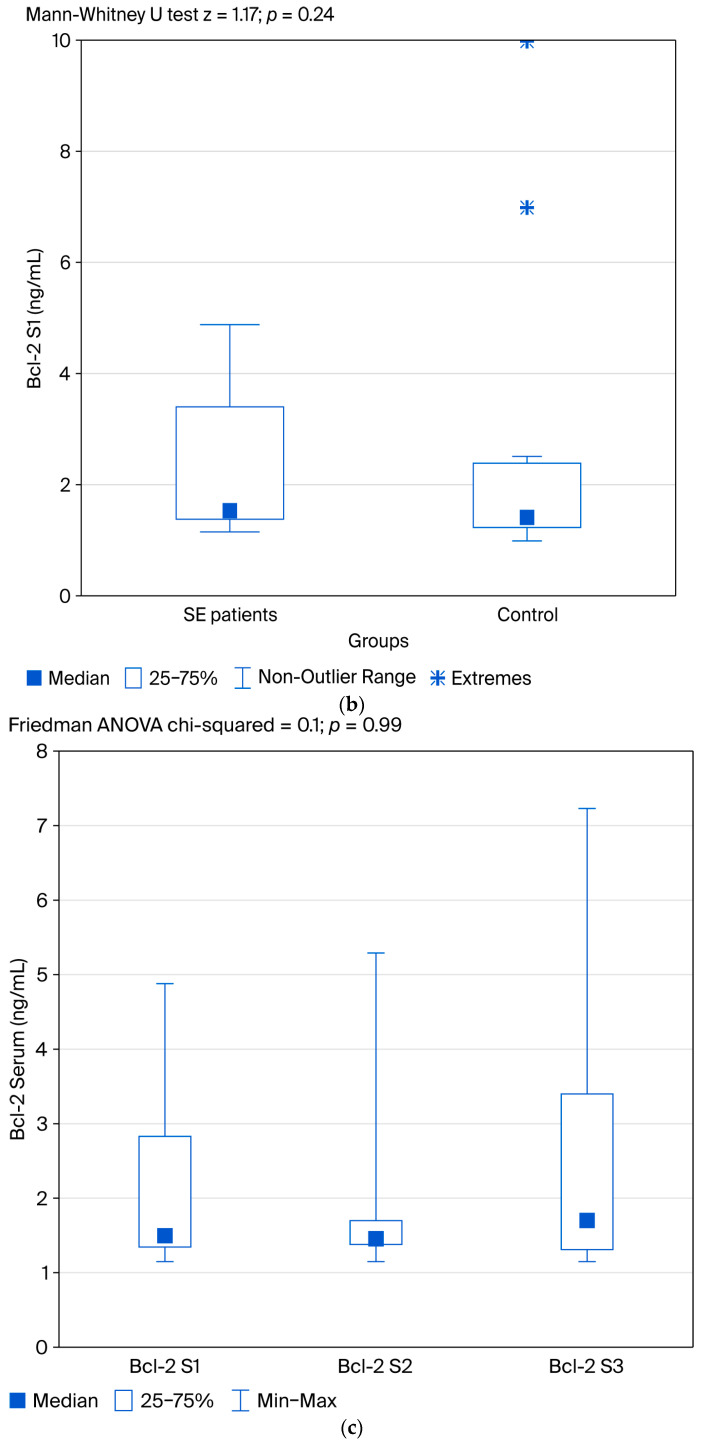
(**a**) Distribution of CSF Bcl-2 in patients with SE and the control group. (**b**) Distribution of serum Bcl-2 in patients with SE and the control group. (**c**) Distribution of serum Bcl-2 at any of the three time points.

The results of the Mann–Whitney U test demonstrated a statistically significant difference in Bcl-2 concentrations in the CSF between patients with SE and the control group (z = 4.1; *p* < 0.001). In contrast, no statistically significant differences were observed in serum Bcl-2 concentrations between the groups at any of the three time points: (Bcl-2 S1: z = 1.17; *p* = 0.24; Bcl-2 S2: z = 1.14; *p* = 0.25; Bcl-2 S3: z = 0.87; *p* = 0.38, respectively) (Figure 2a–c).

**Figure 3:**
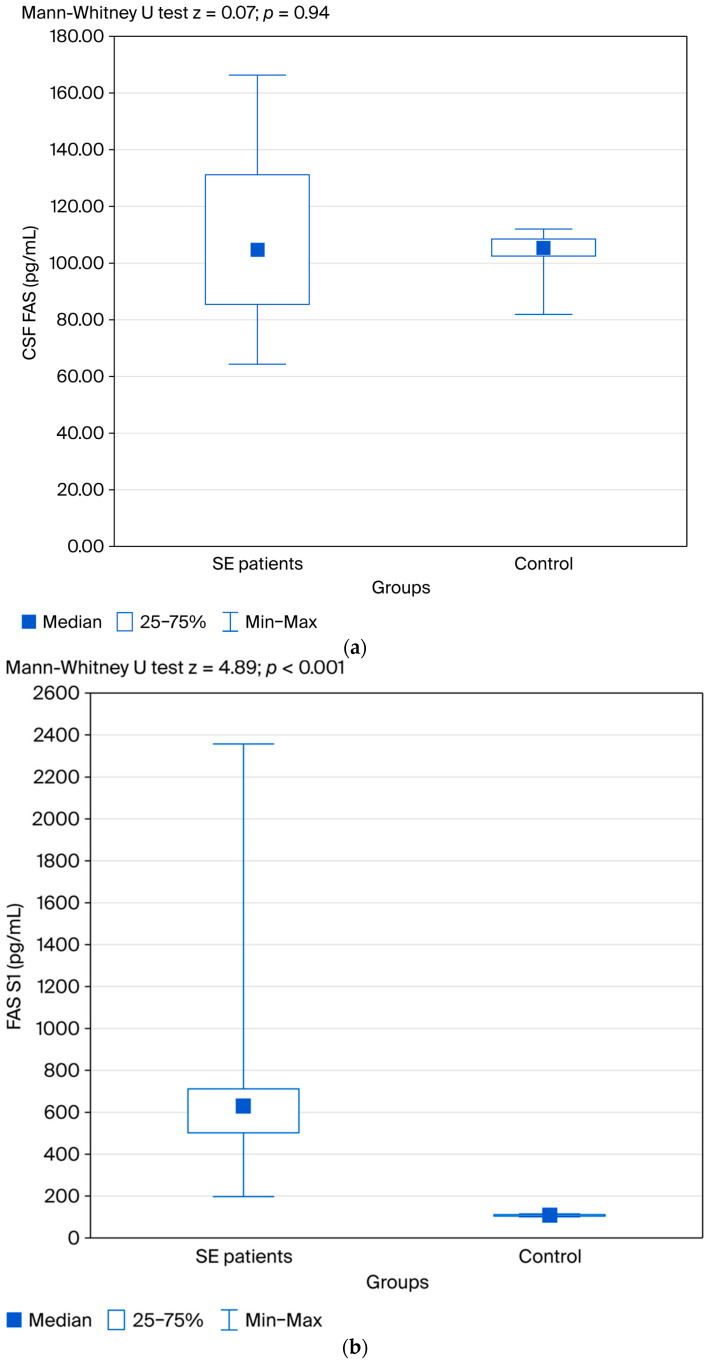

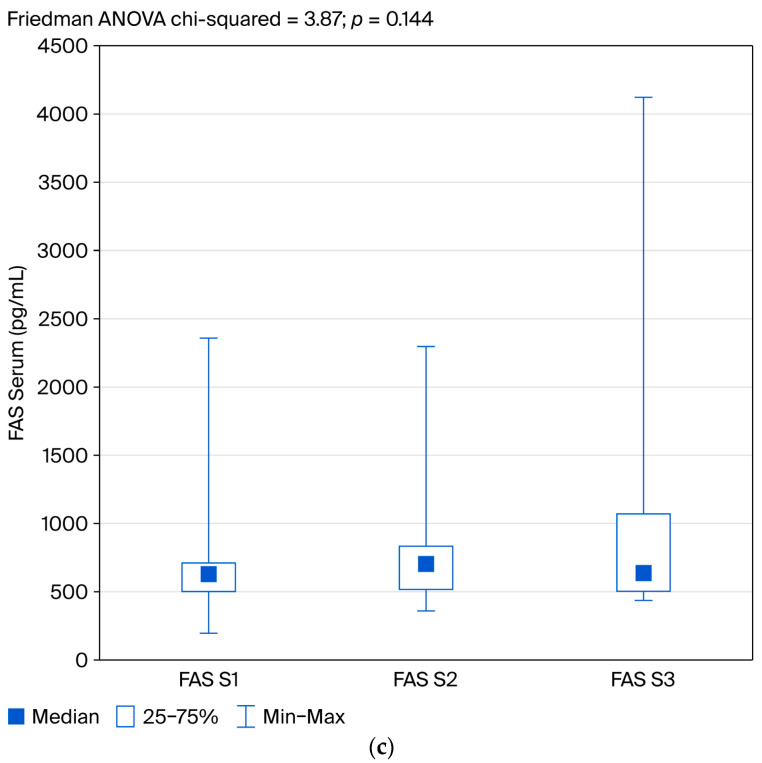
(**a**) Distribution of CSF FAS in patients with SE and the control group. (**b**) Distribution of serum FAS in patients with SE and the control group. (**c**) Distribution of serum FAS in patients at three time points.

Regarding FAS concentrations, no significant difference was observed in the CSF between patients and controls (z = 0.07; *p* = 0.94). On the other hand, serum FAS concentrations were elevated in patients at all three time points compared to the control group, with statistically significant differences: (FAS S1: z = 4.89; *p* < 0.001; FAS S2: z= 4.95; *p* < 0.001; FAS S3: z = 4.89; *p* < 0.001) (Figure 3a–c).

Figure 2a–c indicate the differences in CSF Bcl-2 and serum Bcl-2 concentrations between the groups. Results showed a statistically significant difference in Bcl-2 concentrations in the CSF between patients with SE and the control group. No statistically significant differences were observed in serum Bcl-2 concentrations between the groups at any of the three time points.

Figure 3a–c show the differences in CSF FAS and serum FAS concentrations between the two groups. No significant difference between patients and controls was observed. Serum FAS concentrations were elevated in patients at all three time points compared to the control group, with statistically significant differences.

Statistical analysis using the Friedman ANOVA test did not reveal any statistically significant differences in the values of serum FAS and Bcl-2 markers across the three measurements. For FAS, the Friedman ANOVA chi-square test (N = 18, df = 2) yielded a value of 3.87, with a *p*-value of 0.144, indicating no significant variation. The coefficient of concordance was 0.12, and the average rank correlation (r) was 0.06. For Bcl-2, the Friedman ANOVA chi-square test (N = 18, df = 2) yielded a value of 0.1, with a *p*-value of 0.99, indicating no significant variation.

Comparison between the SE and control groups revealed significantly higher levels of C-reactive protein (CRP), white blood cell count (WBC), CSF lactate, and CSF glucose in patients with SE. Specifically, CRP levels were significantly elevated in the SE group (median 9.1 mg/L, IQR 3.05–30.65) compared to controls (median 2.8 mg/L, IQR 1.1–4.1; z = 2.6, *p* = 0.009). WBC was also significantly higher in SE patients (median 11.0 × 10^9^/L, IQR 8.1–15.10) than in controls (median 7.7 × 10^9^/L, IQR 6.9–9.4; z = 2.09, *p* = 0.036). CSF lactate levels were elevated in the SE group (median 2.50 mmol/L, IQR 2.11–2.64) compared to controls (median 1.6 mmol/L, IQR 1.5–1.8; z = 3.57, *p* < 0.001). Similarly, CSF glucose was higher in the SE group (median 4.50 mmol/L, IQR 4.06–5.24) versus controls (median 3.6 mmol/L, IQR 3.2–4.3; z = 3.21, *p* = 0.001). There was no statistically significant difference in CSF protein levels between the groups (z = 1.46, *p* = 0.146).

Within the group of patients with SE, we compared those with a previous confirmed diagnosis of epilepsy to those without, and found no statistically significant differences in serum or CSF concentrations of either of the two analyzed apoptosis markers. The same applies to subgroup analyses of patients with a previous history of cerebrovascular disease and alcoholism.

Statistical analysis using the Mann–Whitney U test revealed no significant differences in CSF Bcl-2 levels or serum Bcl-2 (S1) between patient subgroups categorized by return to baseline functional status (NORtoB vs. RtoB). Specifically, CSF Bcl-2 levels did not differ significantly between the groups (z = 0.22, *p* = 0.825), and serum Bcl-2 levels also showed no significant variation (z = 0.49, *p* = 0.62). These findings suggest that Bcl-2 concentrations in both CSF and serum are not significantly associated with recovery to baseline functional status in this patient cohort.

Similarly, the difference in CSF Bcl-2 levels between patients with SE who survived and those who died in-hospital was not statistically significant (z = 0.49, *p* = 0.62).

Analysis of serum FAS (S1) levels according to clinical outcome showed a non-significant trend toward higher baseline levels in patients with a fatal outcome. Although this difference did not reach statistical significance (*p* > 0.05), the observed trend may indicate a potential association that warrants further investigation in larger cohorts.

## 4. Discussion

In this study, we demonstrated that cerebrospinal fluid (CSF) levels of Bcl-2 were significantly elevated in patients with status epilepticus (SE), whereas no significant changes were observed in serum. Conversely, serum levels of FAS were elevated, but no significant differences were detected in CSF. These findings suggest that distinct apoptotic mechanisms are activated during SE and support the hypothesis that central and peripheral biological responses to prolonged epileptic activity are not identical.

Current insights into the relationship between prolonged epileptic activity and morphological changes in brain tissue, as indicators of neuronal damage and disruption of their connections, have emerged from the results of long-term scientific research. [6,13,26,27]. Experimental studies have consistently shown that SE activates complex cell death pathways, including the intrinsic mitochondrial pathway, in which Bcl-2 family proteins play a pivotal regulatory role. Murphy et al. demonstrated that Bcl-w, a member of the Bcl-2 protein family, acts as a neuroprotective factor, and its loss increases neuronal vulnerability and accelerates the onset of epileptic activity in experimental SE [28]. Similarly, Henshall et al. reported increased expression of Bcl-2 family proteins in hippocampal tissue from patients with drug-resistant temporal lobe epilepsy, suggesting the activation of anti-apoptotic mechanisms in chronic epileptogenesis [29]. In line with these findings, Toscano et al. showed that although the Bcl-2/BAX ratio is increased in glial and granular neurons of patients with temporal lobe epilepsy, this upregulation does not prevent apoptosis, indicating that intrinsic protective mechanisms may be insufficient to counteract ongoing cell death [30]. Skardaousou et al. further reported elevated serum levels of Bcl-2 and caspase-9 in children and adolescents with idiopathic epilepsy and active seizures, highlighting that systemic markers of apoptosis are also engaged in younger patients and may reflect seizure-related mitochondrial pathway activation [31]. In addition, Xu et al. identified morphological changes consistent with apoptosis in the hippocampus of patients with mesial temporal sclerosis, with a correlation between the expression of Bcl-2, p53, and seizure frequency [32].

Our finding of elevated Bcl-2 levels in the CSF of patients with acute SE may therefore represent a comparable protective neuronal response to acute stress, although the specificity of this marker for neuronal injury remains uncertain. On the other hand, the elevated serum FAS levels we observed are consistent with previous reports in epilepsy patients [33]. For example, El-Hodhod et al. demonstrated increased serum concentrations of both FAS and Bcl-2 in patients with idiopathic epilepsy, although no significant differences were detected between seizure types [34]. Our data, therefore, support the hypothesis that elevated FAS may reflect a systemic response associated with epileptic activity. However, since inflammatory markers (e.g., IL-6, CRP) were not measured in our study, it remains unclear whether elevated FAS reflects apoptotic mechanisms within the CNS or nonspecific systemic inflammation.

Status epilepticus (SE) represents a heterogeneous condition characterized by diverse etiologies, semiology, EEG patterns, and comorbidities, which complicates the formation of uniform study cohorts. This inherent variability presents significant challenges in drawing definitive conclusions and limits the ability to interpret biomarkers such as Bcl-2 in cerebrospinal fluid (CSF) as reliable indicators of neuronal damage in SE. Several key limitations in our study must be acknowledged.

Firstly, the absence of comparator groups with other neurological conditions (e.g., stroke, meningitis) precludes firm conclusions regarding the specificity of CSF Bcl-2 as a marker for neuronal injury in SE. Without such comparative data, it is difficult to determine whether changes in Bcl-2 levels are unique to SE or reflective of broader neuroinflammatory or neurodegenerative processes common to multiple conditions.

Secondly, the relatively small sample size limits the statistical power of our study and restricts our ability to stratify patients based on factors such as etiology, clinical presentation, or SE subtype. This lack of stratification further limits the generalizability of our findings, as the heterogeneous nature of SE means that the biomarker profiles may vary across different patient populations.

Another important limitation is the absence of simultaneous measurements of inflammatory biomarkers, which complicates the interpretation of serum FAS levels. While FAS may reflect apoptotic processes, it could also be indicative of broader systemic immune activation, which could obscure its role specifically within the central nervous system (CNS). Future studies incorporating a broader range of biomarkers, including those associated with systemic inflammation, would be essential to clarify these mechanisms. Moreover, the relatively short observation period of three days for biomarker measurement may have limited the ability to detect longer-term temporal trends. In clinical practice, prognosis in SE is often assessed around day 7, and extending the follow-up period could provide a more accurate understanding of the temporal dynamics of apoptotic processes and their relationship to clinical outcomes.

Methodological constraints related to CSF sampling also need to be considered. Lumbar puncture is an invasive procedure that carries potential risks and discomfort for patients. Consequently, we had to limit the volume of CSF collected to allow for routine diagnostic testing and possible reanalysis with different ELISA dilutions. Additionally, due to contraindications such as ongoing anticoagulant therapy or iatrogenic coagulopathy, it was not feasible to obtain CSF samples from all patients within the same time window after admission. This variability in timing could introduce confounding factors into our analysis. (Supplementary Materials, Tables S1 and S2) Given the invasive nature of lumbar puncture, repeated sampling was deemed unethical and not feasible. As a result, we were unable to perform serial sampling, which could have provided valuable insights into the temporal evolution of apoptotic mechanisms in SE. Finally, although our recruitment strategy aimed to include multiple clinical centers, logistical challenges related to sample storage and processing conditions may have compromised the integrity of samples during transport.

## 5. Conclusions

Our findings indicate that SE activates distinct apoptotic mechanisms within the CNS and periphery, with CSF Bcl-2 possibly reflecting neuronal responses to acute epileptic stress, while elevated serum FAS levels may indicate systemic processes. However, to determine the clinical applicability of these biomarkers, further studies involving larger patient cohorts, additional biomarkers, and comparator neurological disease groups are required.

## Figures and Tables

**Table 1:** Clinical features of patients and the control group. Demographic, clinical, and neurophysiological data for both groups.

	Study Group (18)	Control (15)	
**Age-median**	66	57	Mann–Whitney U z = 2.09, *p* = 0.037
(Q1–Q3)	61–82	37–82	
Min–max	44–84	23–81	
**Sex**			$x^{2}=0.04$ *p* = 0.849 df = 1
Male	9/18	7/15	
Female	9/18	8/15	
**History of previous epilepsy/seizures**	6/18	0	
**History of alcoholism**	6/18	0	
**Cerebrovascular disease**	7/18	8/15	$x^{2}=0.42$ p=0.515,df=1
**History of TBI**	2/18	0	
**SE semiology**			
Focal to BTCS	8/18		
BTCS	4/18		
Unknown to BTCS	6/18		
**SE aetiology**			
Acute symptomatic			
Hyponatremia *	2/18		
Alcohol withdrawal	3/18		
Cessation of ASM	2/18		
Remote	3/18		
Unknown/multifactorial	7/18		
**Outcomes**			
RtoB	8/18		
NO RtoB	6/18		
Death	4/18		
**MR/CT** Periictal changes	3/18		
EEG findings *			
Electrografic	1/18		
Electroclinic	2/18		
LPD	3/18		
IIC	2/18		
Focal/diffuse slowing (RDA)	8/18		
**Estimated SE**			
**duration/treatment**			
<12 h,	8/18		
12–24 h	4/18		
24–48 h	4/18		
>48 h	2/18		

Abbreviations: TBI—traumatic brain injury; ASM—antiseizure medication; BTCS—bilateral tonic–clonic seizure; * hyponatremia in patients with structural changes of the brain caused acute symptomatic SE; RtoB—return to a baseline functional status; NO RtoB—no return to a baseline functional and neurological status; LPD—lateral periodic discharges; IIC ictal–interictal continuum; RDA—rhythmic delta activity; EEG findings * (according to Hirsch LJ et al. American Clinical Neurophysiology Society’s Standardized Critical Care EEG Terminology: 2021 Version. *J Clin Neurophysiol*. 2021 Jan 1;38(1):1–29 [24].

## Data Availability

The original contributions presented in this study are included in the article/Supplementary Material. Further inquiries can be directed to the corresponding author.

## Supplementary Materials

The following supporting information can be downloaded at: https://www.mdpi.com/article/10.3390/jcm14196734/s1.

## Abbreviations

The following abbreviations are used in this manuscript:

SE	Status epilepticus
Bcl-2	B-cell CLL/lymphoma 2, the founding member of the Bcl-2 family
FAS	Fas cell surface death receptor
CSF	Cerebrospinal fluid
ELISA	Enzyme-linked immunosorbent assay
EEG	Electroencephalography
ILAE	International League Against Epilepsy
TLE	Temporal lobe epilepsy
TNFR1	Tumor necrosis factor
DR4 (TRAIL receptor 1)	Death Receptor 4
CSE	Convulsive status epilepticus
GCSE	Generalized convulsive status epilepticus
CNS	Central nervous system
TBI	Traumatic brain injury
ICU	Intensive care unit
CRP	C-reactive protein
IQR	Interquartile range
WBC	White blood cell count
